# A Multifocal Pediatric Papillary Thyroid Carcinoma (PTC) Harboring the *AGK-BRAF* and *RET/PTC3* Fusion in a Mutually Exclusive Pattern Reveals Distinct Levels of Genomic Instability and Nuclear Organization

**DOI:** 10.3390/biology10020125

**Published:** 2021-02-05

**Authors:** Luiza Sisdelli, Maria Isabel V. Cordioli, Fernanda Vaisman, Osmar Monte, Carlos A. Longui, Adriano N. Cury, Monique O. Freitas, Aline Rangel-Pozzo, Sabine Mai, Janete M. Cerutti

**Affiliations:** 1 The Genetic Basis of Thyroid Tumors Lab, Division of Genetics, Department of Morphology and Genetics, Universidade Federal de São Paulo, São Paulo 04039-032, Brazil; l.sisdelli@unifesp.br (L.S.); isabel.cordioli@unifesp.br (M.I.V.C.); 2 Cell Biology, Research Institute of Oncology and Hematology, University of Manitoba, CancerCare Manitoba, Winnipeg, MB R3E 0V9, Canada; aline.rangelpozzo@umanitoba.ca (A.R.-P.); sabine.mai@umanitoba.ca (S.M.); 3 Instituto Nacional do Câncer, Rio de Janeiro 22451-000, Brazil; fvaisman@inca.gov.br; 4 Department of Pediatrics, Irmandade da Santa Casa de Misericórdia de São Paulo, São Paulo 01221-010, Brazil; osmarmonte@uol.com.br (O.M.); carloslongui@msn.com (C.A.L.); 5 Department of Medicine, Irmandade da Santa Casa de Misericórdia de São Paulo, São Paulo 01221-010, Brazil; anamo_cury@hotmail.com; 6 Medical Genetics Service of the Martagão Gesteira Childcare and Pediatrics Institute (IPPMG), Medical School, Universidade Federal do Rio de Janeiro, Rio de Janeiro 21941-912, Brazil; niquecullen@gmail.com

**Keywords:** papillary thyroid carcinoma, pediatric thyroid cancer, nuclear architecture, super-resolution microscope, AGK-BRAF, RET/PTC, biomarkers, genomic instability

## Abstract

**Simple Summary:**

Genetic alterations, such as *RET/PTC* and *AGK-BRAF* fusions, are frequent events in pediatric papillary thyroid carcinoma (PTC). However, their role as prognostic markers in pediatric PTC is still under investigation. In this study, we present a patient harboring three tumor foci with distinct genetic alterations (*AGK-BRAF*, *RET/PTC3* and an absence of canonical alterations) that were investigated for DNA structure and telomere-related genomic instability. These preliminary results highlight that *AGK-BRA*F fusion likely affects nuclear architecture, which might explain a more aggressive disease outcome observed in pediatric PTC cases with *AGK-BRAF* fusion.

**Abstract:**

The spectrum and incidence of gene fusions in papillary thyroid carcinoma (PTC) can differ significantly depending on the age of onset, histological subtype or radiation exposure history. In sporadic pediatric PTC, *RET/PTC1-3* and *AGK-BRAF* fusions are common genetic alterations. The role of *RET/PTC* as a prognostic marker in pediatric PTC is still under investigation. We recently showed that *AGK-BRAF* fusion is prevalent in young patients (mean 10 years) and associated with specific and aggressive pathological features such as multifocality and lung metastasis. In this pilot study, we report a unique patient harboring three different foci: the first was positive for *AGK-BRAF* fusion, the second was positive for just *RET/PTC3* fusion and the third was negative for both rearrangements. To investigate whether *AGK-BRAF* and *RET/PTC3* are associated with genomic instability and chromatin modifications, we performed quantitative fluorescence in situ hybridization (Q-FISH) of telomere repeats followed by 3D imaging analysis and 3D super-resolution Structured Illumination Microscopy (3D-SIM) to analyze the DNA structure from the foci. We demonstrated in this preliminary study that *AGK-BRAF* is likely associated with higher levels of telomere-related genomic instability and chromatin remodeling in comparison with *RET/PTC3* foci. Our results suggest a progressive disruption in chromatin structure in *AGK-BRAF*-positive cells, which might explain a more aggressive disease outcome in patients harboring this rearrangement.

## 1. Introduction

Thyroid carcinoma is the most frequent malignancy of the endocrine system in pediatric patients (≤18 years), where papillary thyroid carcinoma (PTC) is the most common subtype (80–90%) [1,2]. The most prevalent genetic alterations found in pediatric PTC are *RET/PTC* fusions (~41% in sporadic cases, ~58% in radiation-induced cases), where *RET/PTC1* and *RET/PTC3* are the most recurrent rearrangements [1,3]. These rearrangements have been associated with aggressive disease (extrathyroidal extension, lymph node and pulmonary metastasis) [4]. Since they have already been described in benign thyroid tumors, mainly in radiation-induced cases [4], the role of *RET/PTC* as prognostic marker in sporadic pediatric PTC is still unclear.

*AGK-BRAF* [inv (7) (q34)] fusion was originally identified in radiation-exposed PTC cases [5]. Our previous studies have shown that *AGK-BRAF* fusion is a recurrent event in sporadic pediatric PTC cases (19%), and is associated with younger age and pulmonary metastasis of sporadic pediatric PTC [6,7]. Although *AGK-BRAF* has already been associated with the pathogenesis and progression of sporadic PTC cases, its role in genomic instability has not yet been investigated.

Changes in the telomere nuclear architecture and DNA structure remodeling are important features of genomic instability, malignant transformation and aggressiveness [8]. Telomere-related genomic instability plays an important role in cancer and can be used to unmask disease heterogeneity [8]. Moreover, super-resolution microscopy has made it possible to visualize subcellular organization, e.g., the nuclear DNA [9]. In this pilot study, we compared telomere signatures and changes in the chromatin structure of three individual tumor foci that harbored different genetic events (*AGK-BRAF* or *RET/PTC3*) to better understand their role in genomic instability in pediatric PTC.

## 2. Results

### 2.1. Patient Description

A 13-year-old girl with a follicular variant of PTC underwent total thyroidectomy at the hospital of Santa Casa de São Paulo, SP, Brazil. The surgery was followed by four doses of radioiodine (cumulative dose 1150 mCi) treatment. A histological examination showed a bilateral and multifocal tumor with the largest focus measuring 5 × 4 × 2 cm, capsular and angiolymphatic invasion, extrathyroidal extension and lymph node involvement. The patient had five tumor foci, but only three were available for the analysis. Additional findings included lung metastasis at diagnosis and persistent disease during follow-up. There was no family history of thyroid cancer or exposure to radiation. In a previous molecular analysis from a pediatric PTC cohort [10], the patient exhibited the presence of *AGK-BRAF* and *RET/PTC3* rearrangements in two independent foci of the primary PTC, shown in Table 1. A third focus of the primary PTC was also available for analysis, but no genetic alterations were found. Lymph node and lung metastases samples were not available for the analysis.

### 2.2. 3D Analysis of Nuclear Telomere Organization Indicates Increased Telomere-Related Genomic Instability in the AGK-BRAF Positive Focus

**Figure 1:**
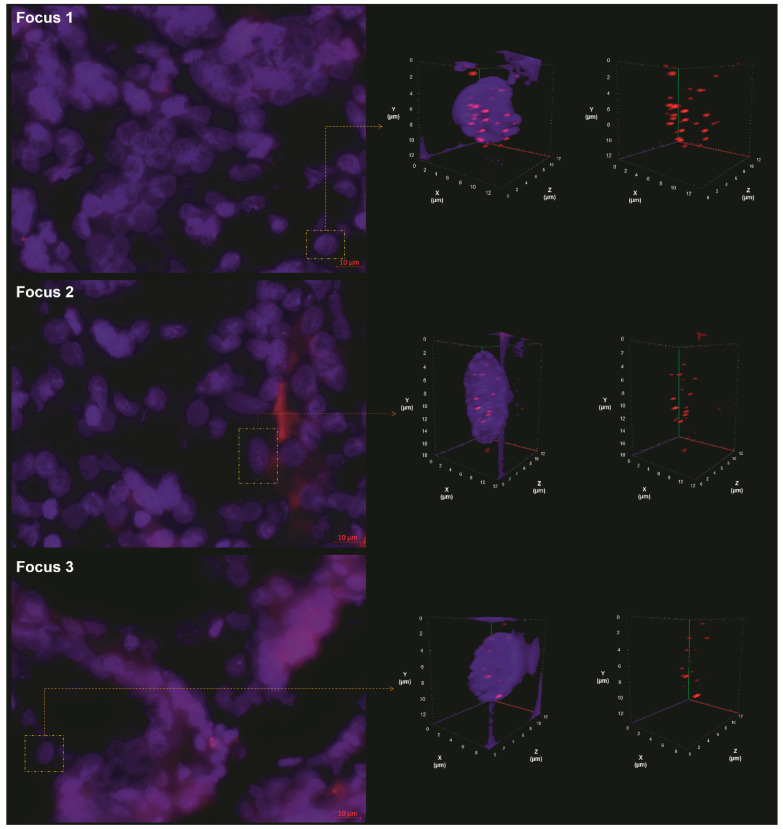
Images from the quantitative fluorescence in situ hybridization (Q-FISH) of the three foci, showing the 2D raw image (**left panel**) and the 3D deconvolved nuclei (**right panel**). The right panel shows the representative 3D nuclear telomere distribution (red signals) with and without the counterstained nucleus (blue). In this figure, we can observe the progressive change of 3D telomere nuclear architecture from focus 1 to focus 3.

Critically short telomeres are hotspots resulting from incorrect recombination, leading to chromosomal instability and malignant transformation [11]. We used 3D quantitative fluorescence in situ hybridization (Q-FISH) to analyze the telomere organization within different foci, as shown in Figure 1. We named as focus 1 the *AGK-BRAF*-positive, as focus 2 the negative for *AGK-BRAF* and *RET/PTC3* fusions (and other investigated alterations [10]), and as focus 3 the *RET/PTC3*-positive, shown in Table 1.

**Figure 2:**
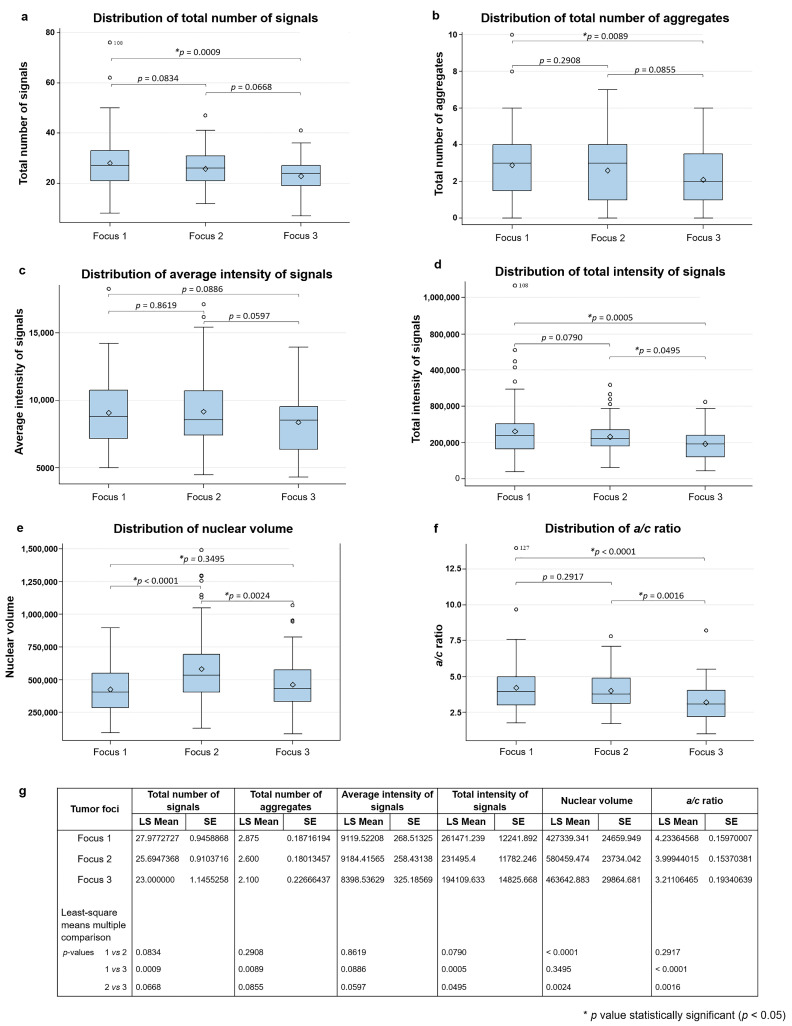
Histogram of the distribution of total number of telomere signals (**a**); total number of telomeric aggregates (TA) (**b**); av-erage intensity of signals (**c**); total intensity of signals (**d**); nuclear volume (**e**) and a/c ratio (**f**). In (**g**), we show the p-values for each comparison made in pairs. LS Mean: least square means. SE: standard error. * p < 0.05.

Telomere parameters comparisons among foci 1, 2 and 3 are shown in Figure 2. Focus 1 showed higher number of telomere signals (*p* = 0.0009) and telomeric aggregates (TA) (*p* = 0.0089) and higher total intensity of signals (*p* = 0.0005) and *a/c* ratio (*p* < 0.0001) in comparison with focus 3, as seen in Figure 2a–d,f. The comparison between foci 1 and 2 showed that focus 2 had higher nuclear volume (*p* < 0.0001), found in Figure 2e. Between foci 2 and 3, focus 2 had higher nuclear volume (*p* = 0.0024) and higher *a/c* ratio (*p* = 0.0016) than 3. Figure 2g shows that no difference was observed in other parameters assessed.

### 2.3. Three-Dimensional Structured Illumination Microscopy (3D-SIM) Measurements Show Significant Changes in DNA Structure in the AGK-BRAF-Positive Focus

**Figure 3:**
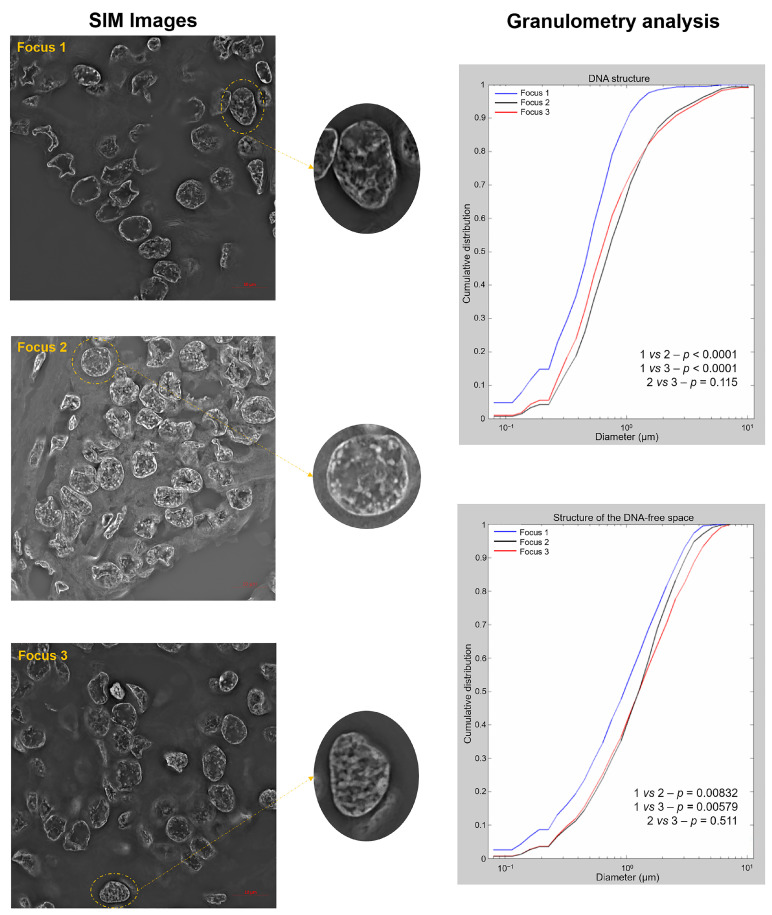
Representative Structured Illumination Microscopy (SIM) images for foci 1, 2 and 3 (**left panel**) and the granulometry comparisons between the foci for DNA structure (**right top panel**) and DNA-poor spaces (**right bottom panel**).

To investigate changes in the DNA structure and the presence of DNA-poor spaces, we used super-resolution imaging and granulometry-based measurements [12]. Granulometry quantified the size distribution of DNA structure and DNA-poor spaces on interphase nuclei. SIM-reconstructed images are presented in Figure 3 (left panel), and the DNA organization, which can be interpreted from the granulometry curves that demonstrate cumulative distributions of granule sizes, is shown in Figure 3 (right panel). Focus 1 showed larger DNA structures and less homogeneous DNA distribution than foci 2 and 3 (*p* < 0.001 for both) (right panel). Moreover, focus 1 presented more DNA-poor spaces than foci 2 and 3 (*p* = 0.00832 and *p* = 0.00579) (right panel), which was illustrated in the nuclei from SIM images (left panel). No difference was observed between foci 2 and 3 (*p* = 0.115, DNA structure; *p* = 0.511, DNA-poor space).

## 3. Discussion

*AGK-BRAF* fusion is a recurrent event in pediatric PTC [6]. However, the mechanism by which *AGK-BRAF* promotes an aggressive phenotype is still unclear. Telomere-mediated genomic instability and chromatin reorganization have been described in tumor cells and correlate with tumor stage and malignant transformation [13]. In this study, we macrodissected three foci of the PTC that were likely to represent the tumor, and examined, for the first time, the effect of different genetic alterations on the telomere organization and chromatin structure. One important conclusion from this study is that representative regions of multifocal PTC may not be entirely informative about overall tumor histology and biology.

Our telomere analysis revealed that focus 1, which harbors *AGK-BRAF*, contained higher numbers of telomeres, total intensity of signals and *a/c* ratio, and more TA when compared to focus 3 with *RET/PTC3* fusion. The presence of more telomeres and telomere fusions in the *AGK-BRAF* focus illustrates genetic aberrations characteristic of cancers, including aneuploidy and gene loss by the breakage-fusion-bridge events initiated by telomere dysfunction. *BRAF* plays an important role in mitosis, mediating proper spindle formation and activation of the spindle assembly checkpoint. Interesting, the BRAF V600E mutation, which leads to constitutive activation of the BRAF kinase, also induces chromosome mis-segregation resulting in aneuploidy [14]. Importantly, BRAF V600E highly activates MAPK pathway in comparison with RET fusion oncoproteins [15]. This is due to unresponsiveness to the negative feedback of activated ERK [15].

Although we did not observe significant differences in the average intensity of telomere signals, total intensity was higher in the focus 1 compared to focus 3. The increased total intensity (a sum value of all intensities in a cell) could have been the result of higher numbers of telomere signals and TA, rather than increased telomere length. Interestingly, focus 2 presented with higher nuclear volume than foci 1 and 3. High nuclear volume or nuclear size or shape are correlated with poor prognosis and progression of some malignancies [16,17]. Cancer cells with high nuclear volumes are often classified as tumors in advanced stages, but the mechanisms behind nuclear volume regulation are still unclear. The *a/c* ratio represents the cell cycle distribution and is correlated with proliferation rates [18]. Increased *a/c* ratio found on focus 1 (*AGK-BRAF*) indicated high proliferation levels. The higher rates in focus 1 could also have been related to the activation of the MAPK pathway induced by the *BRAF* fusion. Although we did not perform Ki-67 staining to compare the *a/c* ratio, it has been shown that the proliferation index measured by Ki-67 staining is extremely similar to the *a/c* ratio measured with TeloView^®^, presenting 98% of concordance between the analysis [19].

Super-resolution imaging can reveal features of carcinogenesis [20]. Our 3D-SIM results showed important changes in the nuclear structure in the focus 1 compared to foci 2 and 3, with more DNA structure and DNA-poor spaces (spaces void of DNA structure). These data suggested that *AGK-BRAF* cells present an increased packaging of the DNA and have more DNA than the other foci. This result aligned with our telomere data, which showed more telomere signals and probably more chromosomes. Previous studies have shown that the number of DNA-poor spaces increases in tumor cells [12,20,21,22], suggesting chromatin remodeling. However, these DNA-free interchromatin areas could be nucleoli, which display the same morphology as DNA-poor spaces and could also be associated with the absence of DNA. We did not perform any staining with antinucleolin antibody or upstream-binding factor (UBF) to differentiate the DNA-poor spaces from nucleoli, a limitation to our study. The granulometry program measures DNA content and absence of DNA signals, not differentiation between DNA-poor spaces and nucleoli. Therefore, the increased number of DNA-poor spaces in the nuclei from focus 1 compared to foci 2 and 3 could be also a consequence of a hypertrophy of nucleoli, which has been correlated with cell proliferation and growth in malignant tumors [23].

Additionally, none of the previously investigated molecular alterations [10] were identified in focus 2. Interestingly, telomere analysis showed no differences between foci 1 and 2 in four telomere parameters (except for nuclear volume). On the other hand, in the 3D-SIM analysis, focus 2 showed a similar structure as focus 3. Further analysis of this focus is necessary to better understand the nature of its genetic background and how it influences nuclear organization and DNA structure.

To conclude, SIM data combined with the 3D telomeric signatures provided a clear discrimination between *AGK-BRAF* and *RET/PTC3* fusions, suggesting distinct levels of genomic instability and nuclear organization. Although additional data is needed to support our results, *AGK-BRAF* fusion is likely associated with a more unstable genetic profile, corroborating with our previously reported data associating this rearrangement with poor prognosis [6].

## 4. Materials and Methods

### 4.1. Patient Samples

Tissue sections (5-µm thickness) from each focus, shown in Table 1, were obtained from formalin-fixed, paraffin-embedded (FFPE) blocks. The selection of the areas from the different tumor foci was performed from Hematoxylin & Eosin (H&E) slides by a pathologist of the Department of Pathology, UNIFESP, as previously described [10]. The Research Ethical Committees from the Universidade Federal de São Paulo and Santa Casa de Misericórdia de São Paulo approved the study (CEP/UNIFESP: 0466/2019).

### 4.2. 3D Telomere Q-FISH, Image Acquisition and Analysis using TeloView^®^ Software Platform

Three-Dimensional Q-FISH was performed following a previously published protocol [24]. In summary, the FFPE tissue sections were deparaffinized by xylene (3 × 10 min), followed by two, 10-min incubations in 100% ethanol. After being air dried, the slides were incubated in 0.2 N HCl (37 °C—30 min) and washed while shaking in ddH_2_O and 2x saline-sodium citrate (SSC) buffer (5 min each) at room temperature (RT). Then the tissues were pre-treated in 1M NaSCN (80 °C—30 min) and washed twice while shaking in 2x SSC (RT—5 min each). The samples were digested using 1 mg/mL pepsin in 0.2 N HCl (37 °C—10 min) and washed twice in 2X SSC (RT—5 min each), while shaking. Then the slides were dehydrated in a series of ethanol (70%, 90% and 100%—5 min each) and air dried. Eight microliters of a telomeric TTAGGG peptide-nucleic acid (PNA) probe conjugated to a Cyanine 3 (Cy3) fluorophore (DAKO, Glostrup, Denmark) were applied onto the tumor areas. Co-denaturation of the DNA and the probe was performed by incubating the slides at 80 °C for 3 min, followed by hybridization, at 37 °C for 2 h, using a HYBrite Denaturation and Hybridization System (Vysis; Abbott Diagnostics, Des Plains, IL, USA). In order to remove the excess nonhybridized probe, the slides were washed twice while shaking in 70% formamide/10 mM Tris-HCl (pH 7.4) (RT—15 min each). Then they were washed while shaking once in 0.1X SSC at 55 °C and twice in 2X SSC/0.05% Tween-20 (RT—5 min each. Lastly, the slides were counterstained using 50 µL of 4′,6-diamidino-2-phenylindole (DAPI) (1 μg/mL) and incubated in the dark for 3 min. Excess DAPI was rinsed with ddH_2_O, and slides were mounted with 22 × 22 mm coverslips using Vectashield mounting medium (Vector Laboratories, Burlington, Ontario, Canada).

One hundred cells from each focus were imaged using a Zeiss AxioImager Z2 microscope equipped with a Zeiss AxioCam MRmm Rev 3 camera (Carl Zeiss Canada Ltd.). The Cy3 filter was used at a constant exposure time (241 ms), while exposure time for the DAPI filter varied. Images were captured in 60 z-stacks at 200-nm intervals to create the 3D images of the cell nuclei. The program AxioVision Release 4.8.2 (Carl Zeiss, Germany) was used for imaging and further imaging processing, using a constrained iterative deconvolution algorithm.

TeloView^®^ v1.03 software program [18] (Telo Genomics Corp., Toronto, ON, Canada) was used to analyze the deconvolved images. TeloView^®^ measures six parameters [18]: number of telomere signals; total intensity of signals; average intensity of signals (telomere length); number of aggregates (cluster of telomeres found in close proximity to each other that, at 200 nm optical resolution, cannot be further resolved as separate entities); *a/c* ratio (cell cycle distribution as being G0/G1, S or G2, according to the position of the telomeres in the cell nuclei; the higher the *a/c* ratio, the greater the proportion of cells in proliferations); and nuclear volume (measured by nuclear DAPI staining in the *x*, *y* and *z* dimensions). The parameters were compared among the three foci using a nested factorial analysis of variance followed by a least-square means multiple comparison. The *p*-value for the overall test of differences between the three foci was indicated by graphical presentations. Chi-square analysis was used to compare the percent of interphase telomere signal intensity as defined quartile cut-offs. A *p* < 0.05 was defined as statistically significant.

### 4.3. 3D-SIM Slide Preparation, Image Acquisition and Analysis

For 3D-SIM, the FFPE samples were deparaffinized using xylene and washed in 1x PBS. Slides were incubated overnight with 10 µg/mL DAPI, in a 37 °C humid chamber and then washed in 1X PBS, air dried and mounted with 18 × 18 mm high performance coverslips (thickness 1 1/2, 0.170 +/− 0.005 mm) (Carl Zeiss Canada Ltd.) using Vectashield mounting medium (Vector Laboratories). We used a Zeiss Elyra PS1 SIM equipped with a Plan-Apochromat 63x/1.40 oil immersion objective, an Andor EM-CCD iXon 885 camera and a 1.6X tube lens (all from Carl Zeiss, Canada) to image 50 cells from each focus. DAPI-stained images were captured using 405 nm laser excitation, a 23-µm diffraction grating and a SR Cube 07 filter cube. Images were acquired at 91-nm intervals between the z-stacks to create the 3D images from the nuclei. Images were reconstructed by ZEN 2012 black edition (Carl Zeiss, Jena, Germany) using the standard settings. Image processing was performed using MATLAB software (MathWorks, Natick, MA, USA). A central z-plane was manually selected and exported as a TIFF file. We used the granulometry program to measure changes in the DNA structure and the presence of DNA-poor spaces with a morphological sieve applied to the error-function clipped images [12]. DNA-poor spaces are DNA-free interchromatin areas that are observed in increased amounts in malignant cells [12]. A statistical analysis was performed by comparing the distributions using two-sided, two-sample Kolmogorov–Smirnov (KS) tests to determine any differences. A *p* < 0.05 was defined as statistically significant.

## Figures and Tables

**Table 1:** Genetic alterations observed in each focus of the pediatric PTC assessed in this study.

	Genetic Alteration
Focus 1	*AGK-BRAF*
Focus 2	No alterations **
Focus 3	*RET/PTC3*

** RAS, BRAF V600E, *RET/PTC1-3*, *AGK-BRAF*, *ETV6-NTRK3* [10].

## Data Availability

The data presented in this study are available in the present article.

## Abbreviations

3D-SIM	3D super-resolution structured Illumination Microscopy
Q-FISH	Quantitative fluorescet in situ hybridization
PTC	Papillary thyroid carcinoma
SIM	Structured illumination microscopy
TA	Telomeric aggregates
